# Re-examination of son-preference based on attitude structure theory under the background of gender imbalance in China

**DOI:** 10.3389/fpsyg.2022.1051638

**Published:** 2023-01-04

**Authors:** Zijuan Shang, Baihui Chi, Ze Liu

**Affiliations:** ^1^School of Marxism, Chang’an University, Xi’an, China; ^2^School of Humanities, Chang’an University, Xi’an, China; ^3^School of Energy and Electrical Engineering, Chang’an University, Xi’an, China

**Keywords:** gender imbalance, son preference, attitude structure theory, public governance, public policy, fertility culture

## Abstract

**Introduction:**

The gender imbalance in China has a long history, and the consequences have begun to emerge, threatening sustainable population development. Against the backdrop of a persistently high sex ratio at birth, the paper introduces the theory of attitude structure to construct an analytical framework explaining the influencing factors of son preference and re-examining the son preference culture.

**Methods:**

Based on the micro-level data from Jiangxi, Guangxi, Guizhou, and Shaanxi Province, this paper adopts a binary logistics model to analyzes the impacts of three cognitive factors on son preference.

**Results:**

The results show that surname inheritance, the utility of elderly care, and the social value in the boy utility evaluation are the main influencing factors in the preference for a son. Social gender assessment, perception of gender imbalance, personal income, education level, health status, and the number of existing children individually correlate with son preference. After comparing the two factors of boy utility evaluation and gender imbalance perception, it is discovered that after China enters a gender-imbalanced society, the perception of gender imbalance effectively reduces the preference for sons, and the son preference is the result of rational choice in people’s fertility process.

**Discussion:**

Based on the above research, the paper proposes policy recommendations at both macro and micro levels from the standpoint of public governance.

## Introduction

China is a populous country. Since 1980, the sex ratio at birth has been high under the influence of the one-child policy and the traditional ideology of son preference (Yang et al., 2009). Although China began to adjust its fertility policy in 2016, the consequences of the gender imbalance caused by the high sex ratio at birth became apparent after nearly 40 years (from 1980 to 2016), especially after 2010. Coupled with its rapid economic development and social transformation, China’s demographic transition has entered a new phase (Li et al., 2014), shifting into a gender-imbalanced society. The gender imbalance that has emerged from China’s population growth is most visibly manifested in the disproportionate number of males to females in all age groups as the millennials and GenZ enter the marriage market.

According to the National Bureau of Statistics, given the average sex ratio at birth of 114.69, there were 352 million male births and 307 million female births between 1980 and 2013. The number of missing female babies in 34 years is between 22.78–35.32 million, based on the usual birth sex ratio of 103–107 (Li and Guo, 2013).

The social problems caused by gender imbalance may be insignificant in childhood. However, in adulthood, several social issues may manifest, including female deficit resulting in marriage squeeze among birth cohorts and in geographical regions (Li et al., 2014), lifelong bachelorhood, increased cost and competitiveness of marriage, threat to family stability, gender imbalance in the labor market, increased barriers to female employment, human trafficking, mercenary and scam marriage, and sexual crimes. The culture of “son preference,” which is the proximate cause of the gender imbalance, will directly affect girls’ survivorship in the early stages of life. Further down the line, it will affect both males and females in the family through marriage squeeze, impacting China’s “universal marriage” and marriage gradient model. Gender imbalance has become a social factor in China in recent times and will continue to be for a long time to come. The array of concerns it poses, such as demographic issues and social problems in culture, economy, and institutions, are closely related to the sustainable development of society.

As indicated by the results of the national sample survey of 1% population in 2015, the sex ratio of the population group aged 0–4 in China is about 116. There is a shared consensus among scholars that son preference is the root cause of the imbalance of sex ratio at birth in China (Yang, 2012). What seems to be the gender preference against females in fertility choices at the individual level is also reflected as gender inequality against women at the societal level, reflecting the institutional loopholes and policy deficiencies of national governance. In the report of the 20th National Congress of the Communist Party of China, General Secretary Xi Jinping highlighted the importance of “adhering to the basic state policy of gender equality and safeguarding the legitimate rights and interests of women and children.” In China, the Party and the government have made it a priority to reduce the prevalent practice of son preference.

Scholars have espoused several theories in explaining the basis for the culture of son preference. First, from an economic perspective, they extended Lebenstein’s “child cost-utility theory” and attributed gender preference in childbirth to people’s rational choice (Jiang and Xiong, 2007; John et al., 2010). Second, from an ethnographic perspective, they believe that son preference is fundamentally an element of patriarchy, patrilineal and patrilocal cultural ideologies. People’s gender preferences in fertility decisions have a basis in cultural systems (Li and Shang, 2012). Third, based on relevant theories of sociology, the desire for son preferences is shaped by personal experience (Yang et al., 2016; Yang, 2017), couple relationship (Yang, 2016), stakeholder attitude (Cai, 2016), and gender role (Li and Zhou, 2018). Fourth, from the perspective of public governance, scholars have analyzed how government actors, through the formulation of policies and programs, such as fertility policies, impact son preference (Yang and Li, 2013; Bi et al., 2015). These theoretical perspectives provide a rich theoretical input for the current study. However, the core element of son preference is a component embedded in individual fertility intention (Gu, 1992), which belongs to the category of social attitude. The existing studies, however, have mostly been conducted from a single theoretical perspective, with a less comprehensive analysis of the factors influencing son preference at the level of individual attitudes.

There is consistency in the conclusions among academics on the fact that son preference culture is the principal cause of gender imbalance, and the measurable effect of culture on reproductive behavior is gender preference (Li and Liu, 2006). Previous gender studies on son preference culture have focused on boy utility and preference degree, and the in-depth analysis shows it is a root cause of gender imbalance. However, in China today, as the effect of the high sex ratio at birth has taken shape, the consequences have gradually surfaced, and the risks have grown markedly (Liu and Li, 2010). The son preference culture has fundamentally altered from the past. Based on attitude structure theory, this thesis builds an analytical framework of the variables associated with son preference, incorporating the relationship between the perception of gender imbalance consequences and son preference. This framework proposes a novel interpretation of son preference that results in gender imbalance and re-examines son preference in China against the backdrop of a gender-imbalanced society. Following the framework, this paper conducts an empirical analysis using micro-personal data from Jiangxi, Guizhou, Guangxi, and Shaanxi in China, the representative provinces with gender imbalance in 2015, and finally makes policy recommendations from the public governance perspective to further alleviate China’s gender imbalance and promote the speed, breadth, and depth of enabling a gender-equal society.

## Framework of son preference psychology influencing factors

Attitude is a structured and relatively stable internal psychological state that individuals hold toward social existence (Le, 2013). Regarding the structure of attitude, M.J. Rosenberg and C.I. Hovland propounded the “theory of three elements of attitude,” which is widely recognized and adopted by scholars. The theory posits that attitude consists of three components: cognition, emotion, and behavior tendency. The cognitive component refers to people’s perception, understanding, and evaluation of the attitude object; the emotional component refers to the individual’s emotion or emotional experience of the attitude object, especially the positive and negative judgments; the behavior tendency component refers to the individual’s internal tendency to react to the attitude object, which is a state of preparation maintained by the individual before acting. The three components interact with and influence each other (Taylor et al., 2004).

Culture is one kind of value concept that influences attitude. The son preference culture in China results from China’s long-standing family and marriage systems that are patrilineal and patriarchal, emphasizing a wife’s subordination to her husband. China’s history as a big agricultural country has resulted in a keen historical preference for sons, which is the cognitive basis for the attitude structure theory. With China’s socioeconomic development, the son preference culture has strengthened the utility of sons in the family under the effect of the one-child policy and then transformed into the habitual practice of selecting male births. Many studies by Chinese scholars on the son preference culture have shown that the fertility culture significantly influences fertility behavior in China, while the culture under the traditional Chinese patriarchal family system has also become an incentive for son preference in fertility behavior (Yang et al., 2011).

**Figure 1:**
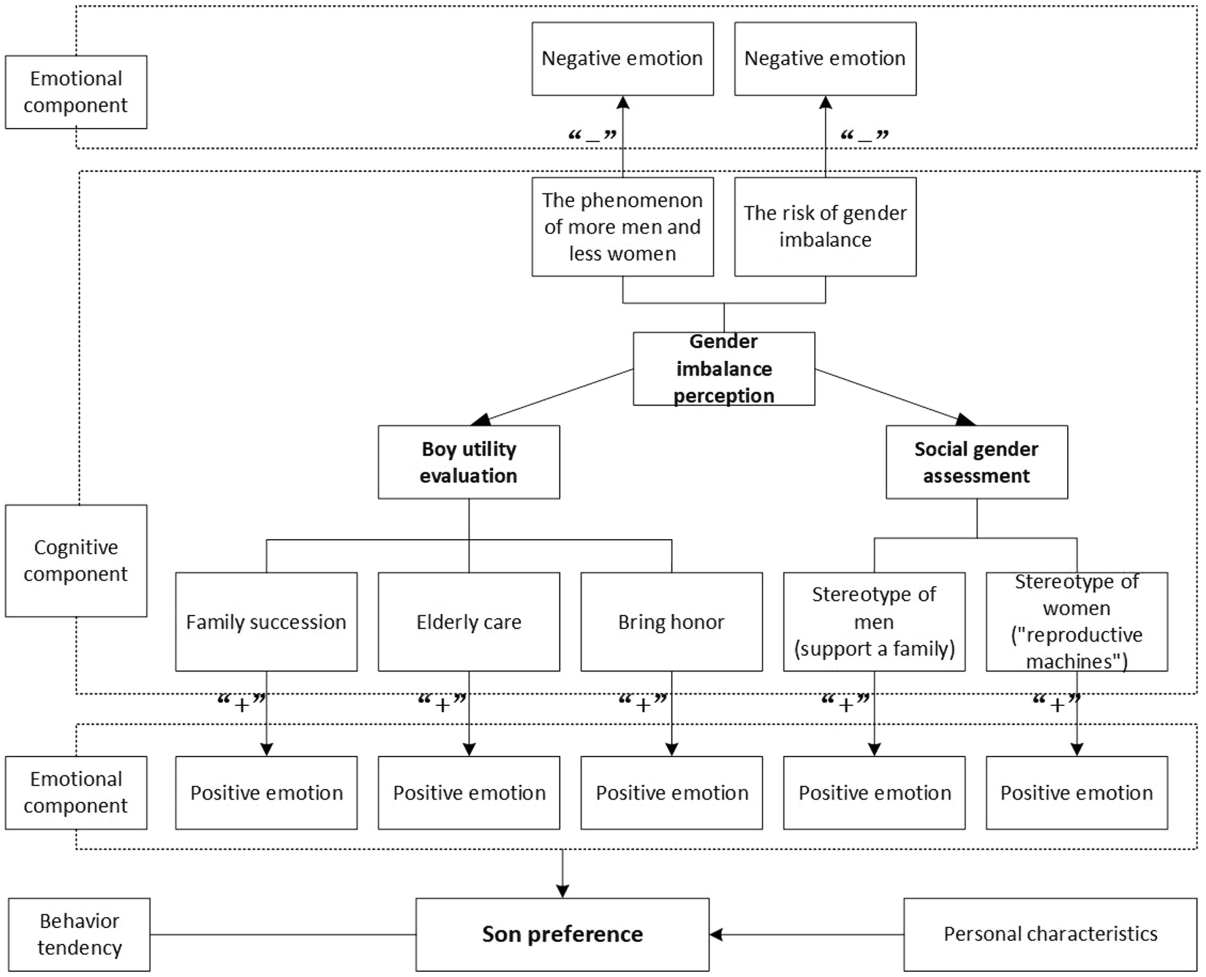
Framework of influencing factors of son preference.

Based on the above theoretical model, the issue of “son preference” studied in this paper is regarded as an individual’s attitude toward the child’s gender in the childbearing process, which has a distinct behavioral tendency and is comprehensively affected by cognitive and emotional components. Therefore, this paper proposes a three-component analysis framework of factors influencing son preference under the background of gender imbalance (see Figure 1).

As a “benefit” factor, boy utility belongs to positive emotion in the emotional utility; that is, it contributes to the formation of son preference. Due to congenital physiological differences, boys and girls differ to a certain extent in terms of utility. In addition, influenced by cultural customs (Guo, 2006) and social institutions (Sheng, 2012), people often believe that sons play far more important roles than girls in terms of effectiveness in relation to family succession, elderly care, and meeting societal needs (Luo, 2003). Family succession is mainly reflected through the transmission of family names and property inheritance.

Gender imbalance is a consequence of son preference, with an emotional distinction between both. In this paper, the perception of gender imbalance is divided into two categories. The first borders on people’s perception of the gender imbalance phenomenon (Jin and Liu, 2009), which is measured by “the increasing number of older unmarried men.” The second relates to people’s perception of the risks brought by gender imbalance (Yang and Li, 2018), which includes the perception of men’s difficulty in getting married, the frequency of malignant events such as mercenary and scam marriage, and women’s safety.

The measurement of social gender assessment here is based on the “fixed effect” of social cognitive theory, i.e., people’s gender stereotypes, which refers to a relatively fixed view of gender (Qian et al., 2013). The stereotype of men is that they must take responsibility for supporting a family and business, while the stereotype of women borders on their perceived role in domestic responsibilities, including caregiving, childbearing and rearing.

People have different attitudes toward the same thing because individuals will be affected by their circumstances when they form their attitudes toward an object. A review of previous research reveals that son preference, as an individual’s attitude toward the child’s gender in the process of childbirth, varies according to individual characteristics like gender, age, and education level. Therefore, in this paper, personal characteristics are included in the analysis framework as the underlying factors when analyzing the factors influencing son preference.

Based on the above analysis, this study uses the “theory of three elements of attitude” to construct a three-component analysis framework of the influencing factors of son preference. In this framework, based on individual characteristics, son preference is affected by three cognitive components: boy utility evaluation, social gender assessment, and gender imbalance perception, as well as their positive and negative emotions. Compared with previous studies, this paper incorporates gender imbalance factors into the analysis framework of son preference from the attitude perspective to examine the new impact of the current demographic situation of gender imbalance on son preference in China (Figure 1).

## Methodology and data

The data comes from the research on *The consequences and countermeasures of China’s high sex ratio at birth* conducted by Xi’an Jiaotong University and the Family Department of the National Health and Family Planning Commission of China in 2015. The survey selected provinces with high gender imbalance in China, including Jiangxi, Guizhou, Guangxi, and Shaanxi. Jiangxi is a province with a consistently high sex ratio at birth, Guizhou is a province with poor economic conditions, Guangxi is a border province, and Shaanxi is a western province. These four provinces are representatives with essential characteristics in the current situation of gender imbalance in China. The survey site selected in this paper also fully reflects the background of gender imbalance in China.

The survey questionnaire adopts the random sampling method. Two counties are randomly selected from each province as the samples, two townships are selected from each county, three administrative villages are selected from each township, and 30 villagers are selected from each village. After excluding the missing value and invalid samples, 1,358 valid samples are obtained. The basic information on the survey objects is shown in Table 1. It should be noted that Jiangxi, with an average sex ratio at birth of 121 in the 2010 census, is the representative of areas with a high sex ratio at birth; Guizhou is also a region with serious consequences of gender imbalance, mainly due to its poor economic conditions and serious loss of women; Guangxi is a border province and is typical of transnational brides and transnational marriages caused by the serious consequences of gender imbalance; Shaanxi also has a relatively high sex ratio at birth and is a typical representative of western provinces.

**Table 1:** Meanings of variables and characteristics of samples.

Type and name of variable		Details and value assignments of variable	Number of samples	Percentage
**Dependent variable**				
It does not matter how many children, as long as there is a son	Binary variable	agree = 1	494	38.03
		disagree = 0	805	61.97
**Independent variable**				
• Boy utility evaluation				
Both boys and girls should take their father’s name	3-category variable	1 = agree	271	20.47
		2 = neutral	255	19.26
		3 = disagree	798	60.27
A daughter should not inherit her parents’ property	3-category variable	1 = agree	913	68.29
		2 = neural	206	15.41
		3 = disagree	218	16.31
One relies on a son to get elderly care	3-category variable	1 = agree	409	30.52
		2 = neutral	434	32.39
		3 = disagree	497	37.09
Having a son will bring social prestige	3-category variable	1 = agree	563	42.46
		2 = neutral	439	33.11
		3 = disagree	324	24.43
• Social gender assessment				
Men must get married	3-category variable	1 = agree	178	13.43
		2 = neutral	300	22.64
		3 = disagree	847	63.92
Women must have children	3-category variable	1 = agree	219	16.4
		2 = neutral	339	25.39
		3 = disagree	777	58.2
A son becomes a live-in son-in-law	3-category variable	1 = agree	713	54.26
		2 = neutral	339	25.8
		3 = disagree	262	19.94
• Gender imbalance perception				
The increasing number of older unmarried men	3-category variable	1 = agree	263	19.91
		2 = neutral	238	18.02
		3 = disagree	820	62.07
It is increasingly difficult for men to get married	3-category variable	1 = agree	283	21.44
		2 = neutral	239	18.11
		3 = disagree	798	60.45
The increasing incidents of mercenary marriage and scam marriage in society	3-category variable	1 = agree	506	38.51
		2 = neutral	211	16.06
		3 = disagree	597	45.43
Women are at great risk of going out alone	3-category variable	1 = agree	306	23.36
		2 = neutral	179	13.66
		3 = disagree	825	62.98
**Control variable**				
Age	Continuous variable		Mean	Standard deviation
			1.73	0.7115
Annual income	3-category variable	1 = <5,000 yuan	589	44.69
		2 = 5,000–10,000 yuan	284	21.55
		3 = more than 10,000 yuan	445	33.76
Health status	3-category variable	1 = good	678	52.48
		2 = general	516	39.94
		3 = bad	98	7.59
Education level	4-category variable	Primary school and below	256	19.36
		Junior high school	650	48.58
		Senior high school	273	20.4
		Junior college and above	156	11.66
Number of existing children	7-category variable	0	33	2.69
		1	401	32.65
		2	672	54.72
		3	111	9.04
		4	8	0.65
		5	2	0.16
		6	1	0.08
Number of samples	*N* = 1,358			

### Methodology

This paper adopts a binary logistics model, and the Stata statistical software was used for data analysis.

First, we built a model of the impact of gender imbalance on boys’ utility and gender judgment. According to the definition of each variable, a multiple linear regression model can be established as follows:

Since the variables are continuous measures, the traditional OLS regression no longer provides an unbiased and valid estimate. Therefore, the Ologit model, a Logit model based on the cumulative distribution, is chosen in the paper. Assuming that the dependent variable is a fixed-order value assigned from 1 to J, the cumulative logit of the dependent variable ≤ *j* and >*j* can be expressed as its basic theoretical model:


(1) $$l_{j}\left(x_{j}\right)=\log\left[\frac{\mathrm{Pr}\left(y_{i}\leq j|x_{i}\right)}{\mathrm{Pr}\left(y_{i}>j|x_{i}\right)}\right]=\alpha_{j}+X\beta$$

In equation (1), X denotes explanatory variables related to boy utility evaluation, containing core explanatory variables and control variables; *β* denotes the coefficient matrix corresponding to X; J denotes the set of categories of boy utility rating, *j*∈*J* = {1, 2}; $\alpha_{j}$ denotes the intercept of the Ologit estimation.

It is worth pointing out that in Ologit estimation, the coefficients of each explanatory variable in the coefficient matrix *β* do not represent the magnitude of the effect of the explanatory variable on the explained variable, but the relative proportion of the occurrence of the 2 cases, *y_i_* ≤ *j* and *y_i_* > *j*, in equation (1), which is called the odds ratio (${\text{oddsratio}}_{i}=e^{-\beta_{i}}$). The odds ratio of the dependent variable from the lower group is $e^{-\beta_{i}}$ times as high as that from the adjacent higher group when the independent variable is increased by one unit.

According to the above settings, the empirical equation used in the paper is


(2) $$\begin{array}{l} l_{j}\left(x_{j}\right)=\log\left[\frac{\mathrm{Pr}\left(y_{i}\leq j|x_{i}\right)}{\mathrm{Pr}\left(y_{i}>j|x_{i}\right)}\right]=\alpha_{j}+\beta_{1}{\text{edu}}_{i}+\beta_{2}{\text{income}}_{i}\\ \;+\beta_{3}{\text{health}}_{i}+\beta_{4}tc14_{i}+\beta_{5}tc17_{i}+\beta_{6}tc110_{i}\\ \;+\beta_{7}tc111_{i}+\sum_{i=1}^{7}\delta_{i}{\text{control}}_{i}\varepsilon_{i} \end{array}$$

In equation (2), ${\text{control}}_{i}$ denotes the demographic and social characteristics and other control variables.

In the second part, we synthesized the influence model of gender imbalance, boy utility and gender judgment on son preference. A multiple linear regression model can be established as follows:


(3) $$y_{i}=b_{0}+\sum_{i=1}^{n}b_{i}x_{i}$$

The dependent variable *y* has *n* predictors (independent) variables *x*_1_, *x*_2_, *x*_3_, … *x_n_*. The relationship between *y* and these *n* independent variables is linear, while *b*_0_， *b*_1_, … *b_n_* are *n + 1* parameters to be estimated.

If there are *k* observation data groups (*x*_*t*1_, *x*_*t*2_, …, *x*_*t*n_; *y*_1_, *y*_2_, …, *y*_k_) *t* = 1, 2, … *k*, the data have the following structure:


$$\begin{array}{l} y_{1}=b_{0}+b_{1}x_{11}+b_{2}x_{12}+\cdots +b_{n}x_{1k}\\ y_{2}=b_{0}+b_{1}x_{21}+b_{2}x_{22}+\cdots +b_{n}x_{2k}\\ \vdots \\ y_{k}=b_{0}+b_{1}x_{n1}+b_{2}x_{n2}+\cdots +b_{n}x_{nk} \end{array}$$

By introducing the variables, the linear equation can be obtained as follows:


(4) $$Y=b_{0}+b_{1}X_{1}+b_{2}X_{2}+\cdots +b_{n}X_{n}$$

The dependent variable *Y* is the function value of the independent variable *X*. *b*_0_ is a constant, where *b*_1_, *b*_2_, … *b_n_* are coefficients corresponding to the values of independent variables *X*_1_, *X*_2_, … *X_n_* in formula (4).

#### Measures

This paper analyzes the impacts of three cognitive factors on son preference through a regression model: boy utility evaluation, social gender assessment, and gender imbalance perception. Moreover, four individual factors: age, personal annual income, health status, and the number of existing children, are included in the model as control variables. The specific variable meanings and sample characteristics are detailed in Table 1.

1. Dependent variable. Son preference refers to people’s particular preference for boys in the process of having children, expressed as a strong desire to give birth to boys (Liu, 2005). The dependent variable, “son preference,” is measured in the questionnaire by the following item “Do you agree that it does not matter how many children you have, as long as there are sons?” If “agree” is selected, it is considered that there is son preference, and the value is coded as 1; selecting the “negative” means that there is no son preference, and the value is coded 0.
2. Independent variable. From the attitude structure theory, this paper focuses on the correlations of three cognitive factors: boy utility evaluation, social gender assessment, and gender imbalance perception on individual fertility preference. The son preference culture directly correlates with boy utility evaluation and social gender assessment, whereas it indirectly correlates with the perception of gender imbalance *via* the link between boy utility evaluation and social gender assessment. According to existing theories of fertility functions, the functions of having boys are primarily measured in terms of economy, culture, and religion. The measurement of boy utility evaluation primarily considers family succession, elderly care utility, and social value based on the relevant questions in the questionnaire. In the variable setting of social gender assessment, the main reference is from the Gender Behavior Scale under the background of Chinese culture (Yang and Li, 2011), and the variables are mainly measured by people’s gender stereotypes. Because China has now entered into a gender-imbalanced society and the marriage squeeze, Vietnamese brides and a group of older unmarried males have garnered widespread attention. The perception of gender imbalance is divided into two parts: the current phenomenon and the consequences to be measured (Shang and Liu, 2019).
3. The collinearity of the independent variables (see Table 2). Because son preference culture and son preference attitudes are interrelated, a multicollinearity analysis was conducted between the variables, examining the variance inflation factor (VIF). The results are presented in Table 3. The model’s mean VIF was 1.32, and the VIF values of the 11 independent variables of boy utility evaluation, social gender assessment, and perception of gender imbalance were all <3. These values were all much lower than the tolerable threshold of 10. As a result, despite the probable conceptual overlap among the variables, the overall model did not have substantial collinearity issues.

**Table 2:** Results of multicollinearity test.

Variable	tb14	tb16	tb11	tc120	tb19	tb110	tc115	tc14	tc17	tc110	tc111
VIF	1.32	1.13	1.27	1.05	1.77	1.82	1.05	1.32	1.27	1.27	1.28
1/VIF	0.759	0.884	0.79	0.951	0.564	0.549	0.956	0.76	0.79	0.787	0.784
Mean value of VIF	1.32										

**Table 3:** Logistic model results for son preference.

Variable			Model 1	Model 2	Model 3	Model 4	Model 5
Personal characteristics	Age		0.021^*^	0.028^**^	0.027**	0.014+	0.013
	Education level	Junior high school	−0.274	−0.352*	−0.506**	−0.431*	−0.444*
		(primary school and below)					
		Senior high school	−0.438+	−0.825***	−0.965***	−0.609**	−0.615**
		(primary school and below)					
		Junior college and above (primary school and below)	−0.184	−0.551+	−0.748**	−0.455+	−0.471+
	Annual income	10,000–20,000 yuan	0.257	0.039	0.216	0.323+	0.264
		(<10,000 yuan)					
		More than 20,000 yuan	0.369*	0.221	0.361*	0.484	0.497**
		(<10,000 yuan)					
	Health status	General (good)	−0.492***	−0.326*	−0.348*	−0.363*	−0.334*
		Bad (good)	0.027	0.261	0.165	0.167	0.161
	Number of existing children		0.234*	0.267**	0.258**	0.258*	0.274*
Boy utility evaluation	Both boys and girls should take their father’s name	Neutral (disagree)	0.121*			0.157	0.206
		Agree (disagree)	0.143*			0.151	0.083
	A daughter should not inherit her parents’ property	Neutral (disagree)	0.525**			0.509**	0.665**
		Agree (disagree)	0.634***			0.629**	0.557**
	One relies on a son to get elderly care	Neutral (disagree)	0.608**			0.601**	0.651**
		Agree (disagree)	1.322***			1.316***	1.309***
	Having a son will bring social prestige	Neutral (disagree)	−0.057			0.238	0.229
		Agree (disagree)	0.606***			0.602***	0.706***
Social gender assessment	Men must get married	Neutral (disagree)		0.081			−0.033
		Agree (disagree)		0.010+			−0.169
	Women must have children	Neutral (disagree)		−0.671*			−0.935**
		Agree (disagree)		0.572*			0.233
	A son becomes a live-in son-in-law	Neutral (disagree)		−0.345*			−0.094
		Agree (disagree)		−0.551**			−0.605**
Gender imbalance perception	The increasing number of older unmarried men	Neutral (disagree)			−0.500*	−0.546*	−0.410+
		Agree (disagree)			−0.152	−0.203	−0.161
	It is increasingly difficult for men to get married	Neutral (disagree)			0.046	0.236	0.218
		Agree (disagree)			−0.109+	−0.129*	−0.165
	The increasing incidents of mercenary marriage and scam marriage in society	Neutral (disagree)			0.186*	0.154+	0.225+
		Agree (disagree)			0.029*	0.04	0.124
	Women are at great risk of going out alone	Neutral (disagree)			−0.356*	−0.384+	−0.220+
		Agree (disagree)			0.016	−0.048	−0.024
-2LL			1193.8	1231.1	1258.8	1106.6	1040.1
Pseudo *R*^2^			0.138***	0.103***	0.061***	0.148***	0.177***
Number of samples			1,358	1,337	1,326	1,299	1,235

What lies in the blankets are the references; ***p < 0.001, **p < 0.01, *p < 0.05, +*p* < 0.1.

All the items were measured with a five-point Linkert scale from “strongly disagree” to “strongly agree” and are treated as three-category variables with values of 1, 2, and 3 to facilitate analysis. In addition, individual characteristic factors such as age, personal annual income, education level, health status, and the number of existing children are included in the regression model as control variables.

## Results

In this paper, the binary logistic model is used to analyze “son preference,” and five models are constructed: model 1, model 2, and model 3 are the correlations of three factors on son preference: boy utility evaluation, social gender assessment, and gender imbalance perception. Model 4 is the regression result after taking into account both boy utility evaluation factors and gender imbalance perception factors together with the control variables. Model 5 comprehensively considers three kinds of individual cognitive factors: boy utility evaluation, gender imbalance perception and social gender assessment, and compares and analyzes the correlations of different variables on son preference (see Tables 3, 4).

**Table 4:** Correlation of gender imbalance perception with boy utility evaluation and social gender assessment.

Variable		Model 6	Model 7	Model 8	Model 9	Model 10	Model 11	Model 12
Boy utility evaluation		Both boys and girls should take their father’s name	A daughter should not inherit her parents’ property	One relies on a son to get elderly care	Having a son will bring social prestige	Men must get married	Women must have children	A son becomes a live-in son-in-law
Social gender assessment								
Personal characteristics	Age	0.0160*	0.0179* (0.00720)	0.0347***	0.00957	0.0193**	0.0232***	−0.000387
		−2.34		−5.44	−1.56	−2.78	−3.5	(−0.06)
	Education level	−0.271***	−0.299*** (0.0801)	−0.344***	−0.062	−0.320***	−0.221**	−0.087
		(−3.82)		(−5.08)	(−0.94)	(−4.38)	(−3.14)	(−1.25)
	Annual income	−0.0871	0.0209 (0.0772)	−0.142*	0.0299	0.0542	0.0723	0.0694
		(−1.20)		(−2.09)	−0.45	−0.73	−1.01	−0.99
	Health status	−0.212*	−0.0510 (0.106)	−0.113	0.149	−0.0634	−0.0887	0.238*
		(−2.13)		(−1.20)	−1.59	(−0.61)	(−0.89)	−2.46
Perception of gender imbalance	The increasing number of older unmarried men	−0.00603	−0.0474	0.0238	0.14	0.106	0.0375	−0.072
		(−0.07)	−0.092	−0.29	−1.69	−1.16	−0.43	(−0.86)
	It is increasingly difficult for men to get married	0.205*	0.129 (0.0894)	0.051	0.0755	0.138	0.125	−0.00453
		−2.49		−0.65	−0.96	−1.63	−1.52	(−0.06)
	The increasing incidents of mercenary marriage and scam marriage in society	−0.132	−0.0404 (0.0781)	−0.143*	0.124	−0.182*	−0.202**	0.277***
		(−1.74)		(−2.09)	−1.8	(−2.34)	(−2.73)	−3.84
	Women are at great risk of going out alone	0.287***	−0.128 (0.0840)	0.0669	0.0485	0.247**	0.238**	0.0069
		−3.56		−0.89	−0.64	−3.03	−3	−0.09
Constant cut1		−0.980*	−0.980*	−0.59	1.180**	−1.032*	−0.671	0.883
		(−2.07)	(−2.07)	(−1.32)	−2.65	(−2.11)	(−1.43)	−1.93
Constant cut2		0.0059	0.0059	0.847	2.632***	0.298	0.673	2.144***
		−0.01	−0.01	−1.89	−5.84	−0.61	−1.44	−4.65
Sample size		1,136	1,136	1,146	1,151	1,142	1,144	1,140

What lies in the blankets are the references; ***p < 0.001, **p < 0.01, *p < 0.05, +*p* < 0.1.

### The influence of individual characteristics on son preference

The statistical results of the five models show that the individual characteristics of the respondents have a significant impact on son preference. The regression results of age and son preference are consistent with the previous research conclusions, showing that older people have a stronger son preference (Liao et al., 2012). The results also reflect that people with more children have a stronger preference for boys. The reason may be that people often increase the probability of giving birth to boys by increasing the number of children (Qiao, 2004), likely to further compound the problem of the high sex ratio at birth in China for a long time. The analysis of education level is also consistent with the original research results. The higher the education level, the weaker the son preference, indicating that improving people’s education level can weaken the culture of son preference (Dou et al., 2019). In the result from the model, the education level of senior high school is the dividing point affecting son preference.

In the regression results of individual annual income and son preference, respondents with an annual income of “more than 20,000 yuan” have a stronger son preference than those with an annual income of “>10,000 yuan.” But respondents with an income of “between 10,000 and 20,000 yuan” have no significant son preference compared with low-income earners, indicating an “inverted U-shaped” relationship between son preference and income. This validates the conclusions in previous research studies (Yang and Gillian, 2012). It can be explained by the high cost of having a boy and the change of orientation likely to be promoted by the increased income: low-income people have difficulties affording the cost of giving birth to boys, while high-income people have a weak gender preference in fertility decisions. In addition, health status has a significant relationship with son preference in all five models, indicating that when individuals are in average health, it will significantly inhibit the son preference.

### The influence of boy utility evaluation on son preference

According to the results in Model 1, among the three types of utilities, the utility of elderly care and property inheritance have more significant correlations with son preference, followed by social value, indicating the dominance of economic utility in son preference (Liu, 2006). Moreover, it also shows that the “face culture” is a significant factor in shaping son preference (Li et al., 2003). In the effectiveness of family succession, the role of sons in the continuation of the family name also has a significant impact on gender preference. This is consistent with the finding of Li, 2012, who reported that surname transmission is central to family patriarchy and plays a vital role in the preference for male children.

Model 4 is a comprehensive analysis of boy utility evaluation and perception of gender imbalance risk, which play opposite emotional roles in shaping son preference, with the former being a “benefit” and the latter a “risk.” The results show that the significance and coefficients of some variables were attenuated, but in comparison, boy utility evaluation has a more significant impact on son preference. After the variable of gender imbalance perception is added, people’s preference for boys will change when they perceive that the current society is in a state of gender imbalance, which poses a risk. However, when the utility of boys is deeply rooted in people’s minds and widely recognized, factors such as “risk” and “cost” play little role, which also shows that people’s gender preference in fertility is a rational choice based on cost–benefit (Yang, 2014).

In model 5, after incorporating social gender assessment and perception of gender imbalance, the impacts of the utility of elderly care and social value on son preference remain significant, indicating that gender preference arises more from people’s need for boys’ utility (Tao, 2012). The need fundamentally derives from social existence, i.e., various explicit systems and implicit rules currently operate in society, such as family patriarchy, child-rearing, and succession of the family line.

### The influence of gender imbalance perception on son preference

Model 3 is a regression analysis of the impact of gender imbalance perception on son preference. It is found that the perception of the consequences of gender imbalance has a significant association with son preference. One influence is “an increasing number of older unmarried men,” which belongs to the phenomenon of gender imbalance. The other is the consequence of the marriage squeeze caused by gender imbalance, i.e., “it is increasingly difficult for men to get married.” It shows that when people understand and perceive the risks brought by gender imbalance, their son preference will be affected accordingly due to the instinctive reaction of “seeking advantages and avoiding disadvantages.” The problem that also has a significant impact on son preference is “the increasing number of cases of mercenary and scam marriages in society,” and the model results indicate that people who are “neutral” on this problem have a stronger preference for boys. The preliminary analysis suggests that people who show “indifference” on this issue are not concerned with incidents such as “mercenary marriage” and “scam marriage” and do not realize the harm of such events, which fundamentally reflects an incorrect understanding of the purpose, significance and value of marriage, resulting in a stronger son preference. In addition, people who perceive that “women are at great risk of going out alone” have a significant decline in their son preference, indicating that women’s safety problems caused by gender imbalance inhibit people’s son preference to a certain extent.

Whether in model 4, which incorporates boy utility evaluation factors, or in model 5, which combines boy utility evaluation, gender imbalance perception and social gender assessment, the “increasing incidents of mercenary and scam marriages in society” and “women are at great risk of going out alone” that measure gender imbalance perception have always had a significant impact on son preference. The former is a remarkable embodiment of son preference in specific groups, and the latter is strong proof that gender imbalance exerts a countervailing influence on son preference. While both can have an inhibiting relationship with people’s preference for boys, the two issues show that the consequences of the gender imbalance in China are already visible, and problems such as gender imbalance (Yang et al., 2017) and the marriage squeeze pose a tremendous risk to the health of our society (Shang and Liu, 2019). Meanwhile, after incorporating the variables of gender imbalance perception, the overall explanatory degree of the son preference model has been significantly improved. Compared with model 1 and model 2, the explanatory power of model 4 and model 5 has been increased by more than half, indicating that people’s perception of gender imbalance has an essential impact on son preference.

### The influence of gender judgment on son preference

Model 2 is a regression analysis of the impact of gender evaluation on son preference. The results show that the two problems, “men must get married” and “women must have children,” influence son preference significantly. To a certain extent, the two reflect boys’ utility. The former is the recognition of men’s “head of the family” status, the mission of supporting a family and the function of family succession, and the latter is a description of the stereotype of women as “reproductive machines,” indicating that people’s inherent gender judgment will lead to the formation of son preference. The “son becomes a live-in son-in-law” also has a significant impact on son preference, and the impact coefficient is negative, indicating that with the change in people’s gender stereotypes, a more tolerant gender environment will weaken people’s son preference culture.

## Conclusion and discussion

Based on the analysis framework of the influence of son preference, this paper selects the personal data of four typical provinces in China to conduct an empirical study. From the analysis results, this paper not only verifies the theoretical hypotheses and supports the conclusions of the existing research on the causes of son preference, but also has some new findings because a new perspective of gender imbalance perception was incorporated.

First, the inheritance of surnames is the core of family patriarchy, which lays the cultural and institutional foundation of son preference. In the model results, compared with the influence of property inheritance on son preference, the correlation of surname inheritance is more significant. The succession of surnames is the core of family patriarchy, and in the traditional concept, only men can “carry on the family line,” which also constitutes the cultural ground for shaping son preference.

Second, social gender assessment is an important factor affecting son preference. The results of regression analysis on gender evaluation and son preference show that the two issues among gender evaluation factors have a significant impact on son preference. Not only does this supports the relevant research on analyzing son preference from a gender perspective, but also shows that gender is an important perspective in the research on the development mechanism of son preference, which deserves further exploration.

Third, the perception of gender imbalance, particularly the perception of marriage squeeze, inhibits the internalization of son preference at one level. Traditional Chinese culture values marital status, and due to China’s long-standing high sex ratio at birth, a surplus of males has begun to enter the marriage line, progressively pushing the marriage squeeze into the public sphere. The consequences of gender imbalance, such as high bride prices and Vietnamese brides, have also begun to surface, which has deepened the public’s perception of a gender-imbalanced society. The model demonstrates a rising perception of older single males, mercenary marriage, and scam marriage phenomena, which is strongly correlated with people’s desires for boys, and it implies that we should act to address the consequences of gender imbalance.

Fourth, boy utility evaluation dominates the three types of individual cognitive factors affecting the formation of son preference, and economic utility plays a decisive role in shaping son preference. After the other two types of cognitive factors are successively incorporated into the model, the impact of boy utility evaluation on son preference remains significant, and the elderly care utility has always maintained a highly significant impact in the five models, which shows that the concept of “raising children for elderly care” is widespread and deep-rooted in the hearts of Chinese people, and that economic motivation is the root cause of son preference.

Fifth, individual characteristics have an important impact on son preference, and reflect the strength of son preference to a certain extent. Among them, personal income and health status are significant influencing factors of son preference, while the number of existing children reflects the degree of son preference of individuals and their families, explaining the strength of son preference. Education level can affect people’s son preference to a certain extent, and improving the average education level can weaken the son preference culture.

This study, like most others in the field of son preference research, explores the relationships between the son preference culture and pertinent variables from an individual viewpoint, so it has a micro-level limit. With the adjustment of China’s fertility policy, a universal three-child fertility policy has now been implemented, and socio-cultural, economic, and political changes have taken place. Because China’s long-standing urban–rural dichotomy structure and regional distinctions have resulted in urban–rural disparities, the gender imbalance has regional and urban–rural variations (Li et al., 2011). Consequently, future research should consider the external social environment’s potential impact on China’s culture of son preference, and the distribution of the study sample should be widened. The method used to generate a multi-layer structural model can be utilized to incorporate the changes in the external macro-environment into the son preference model developed in this paper, combining the macro and micro viewpoints to explain the most recent changes in son preference in China.

## Data availability statement

The original contributions presented in the study are included in the article/supplementary material, further inquiries can be directed to the corresponding author.

## Ethics statement

Ethical review and approval was not required for the study on human participants in accordance with the local legislation and institutional requirements. Written informed consent from the participants was not required to participate in this study in accordance with the national legislation and the institutional requirements.

## Author contributions

ZS was responsible for the conceptualization of the thesis framework and wrote the original draft. BC wrote the English manuscript and contributed to the editing. ZL analyzed the data. All authors contributed to the article and approved the submitted version.

## Funding

This research was supported by the National Social Science Fund of China (Grant No.19ARK004) and Social Science Fund Project of Shaanxi Province (Grant No.2021F002).

## Conflict of interest

The authors declare that the research was conducted in the absence of any commercial or financial relationships that could be construed as a potential conflict of interest.

## Publisher’s note

All claims expressed in this article are solely those of the authors and do not necessarily represent those of their affiliated organizations, or those of the publisher, the editors and the reviewers. Any product that may be evaluated in this article, or claim that may be made by its manufacturer, is not guaranteed or endorsed by the publisher.
